# Who you live with and what you duet for: a review of the function of primate duets in relation to their social organization

**DOI:** 10.1007/s00359-023-01689-9

**Published:** 2024-01-29

**Authors:** Chiara De Gregorio, Daria Valente, Valeria Ferrario, Filippo Carugati, Walter Cristiano, Teresa Raimondi, Valeria Torti, Cristina Giacoma, Marco Gamba

**Affiliations:** 1 Department of Life Sciences and Systems Biology, University of Torino, Turin, Italy; 2 Environment and Health Department, Italian National Institute of Health, Rome, Italy

**Keywords:** Pair living, Group living, Mate defense, Territorial defense, Loud call, Bird song

## Abstract

Duets are one of the most fascinating displays in animal vocal communication, where two animals fine-tune the timing of their emissions to create a coordinated signal. Duetting behavior is widespread in the animal kingdom and is present in insects, birds, and mammals. Duets are essential to regulate activities within and between social units. Few studies assessed the functions of these vocal emissions experimentally, and for many species, there is still no consensus on what duets are used for. Here, we reviewed the literature on the function of duets in non-human primates, investigating a possible link between the social organization of the species and the function of its duetting behavior. In primates and birds, social conditions characterized by higher promiscuity might relate to the emergence of duetting behavior. We considered both quantitative and qualitative studies, which led us to hypothesize that the shift in the social organization from pair living to a mixed social organization might have led to the emergence of mate defense and mate guarding as critical functions of duetting behavior. Territory/resource ownership and defense functions are more critical in obligate pair-living species. Finally, we encourage future experimental research on this topic to allow the formulation of empirically testable predictions.

**Supplementary Information:**

The online version contains supplementary material available at 10.1007/s00359-023-01689-9.

## Introduction

Animals communicate through a great variety of sounds. From the chirps of birds to the howling of wolves and the buzzing of cicadas, many animals rely on the acoustic channel to convey information that facilitates interactions with conspecifics. Depending on the position of the intended receiver and the call functions, vocal signals have different features in terms of duration, frequency, and intensity (dB SPL; Gamba et al. 2015; Riondato et al. 2021). Contact calls between two animals foraging together are usually less intense than calls aimed at distant receivers, such as neighboring groups during territorial confrontations or separated social partners (Salmi and Doran-Sheehy 2014; Bonadonna et al. 2020). Vocalizations used during the latter context have been defined as long-distance, long-range, or ‘loud calls’ (Mitani and Stuht 1998). These seem to serve different functions, mediating communication between intergroup and intragroup. For example, gorillas emit loud hoots to reduce distance among separated social partners (*Gorilla gorilla*; Salmi and Doran-Sheehy 2014), while gray-cheeked mangabeys use loud calls for intergroup spacing (*Lophocebus albigena*; Waser 1975), and maned wolves use long-distance calls to maintain relationships with distant individuals (*Chrysocyon brachyurus*; Ferreira et al. 2022).

**Fig. 1:**
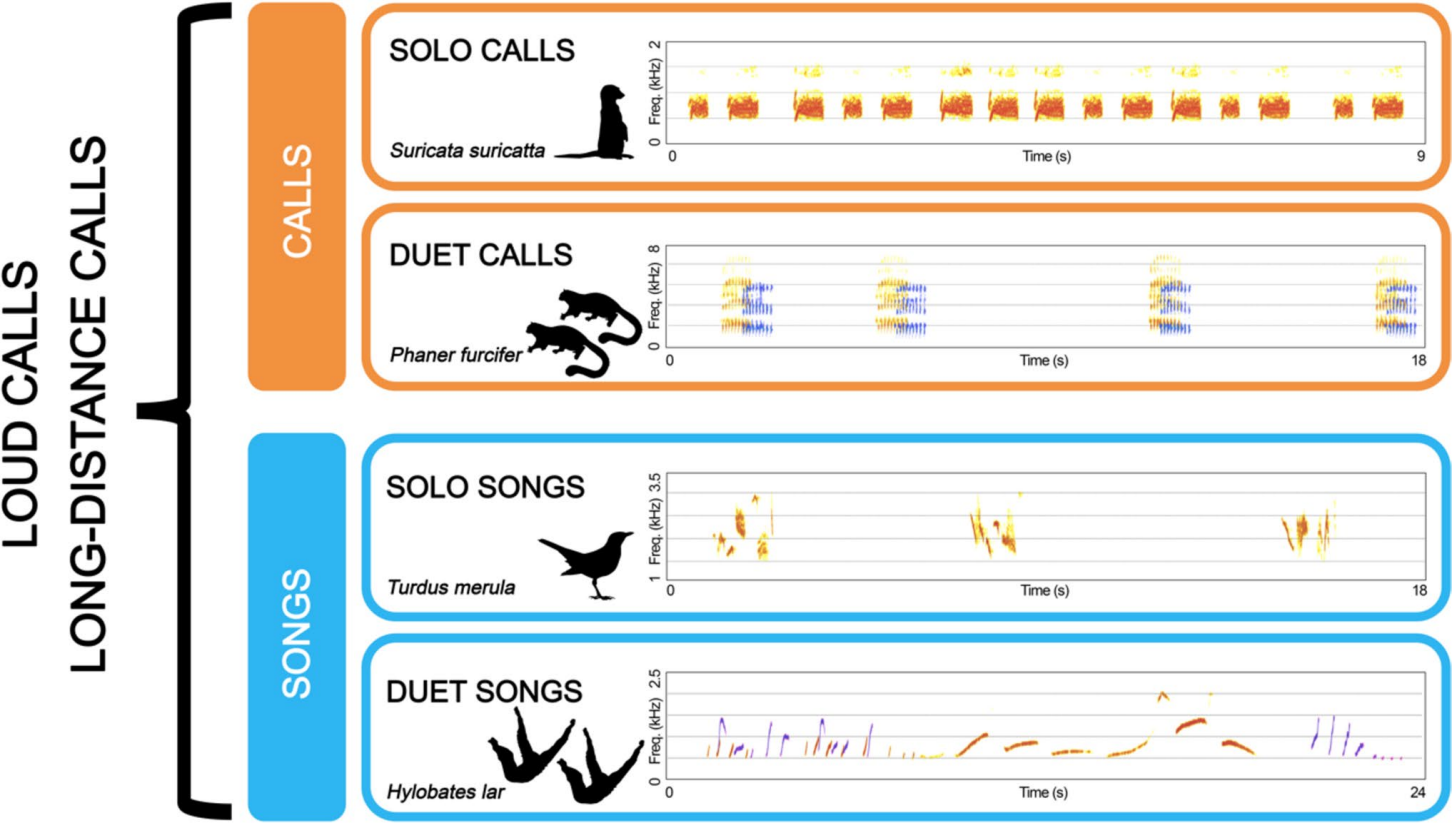
Spectrogram of the types of vocalizations that can be considered as loud calls or long-distance calls. Different colors represent sounds emitted by different individuals

Nevertheless, long-distance calls or loud calls are very general terms that can contain a variety of different types of vocalizations, depending on the structure of the signal, such as songs and calls, and the number of individuals involved, such as duet calls and solo calls (Fig. 1). This review aims to focus on duetting behavior, a type of loud/long-distance call in which two animals fine-tune the timing of their emissions to create a coordinated signal. Despite duets being one of the most fascinating displays of animal vocal communication, there is no consensus on their functions. Therefore, we reviewed the available literature on the function of duets in non-human primates, investigating a possible link between the social organization of the species and the function of its duetting behavior.

## Duets

### What are duets?

Duets represent a special type of loud call (or long-distance call). Duets can be considered a form of loud call because of their high intensity. Although many works on duetting animals do not report the intensity of the vocal emission, when this information is available, it shows that duets can be loud. For example, in primates, siamang calls have a mean maximum intensity of 99 dB SPL (*Symphalangus syndactylus*, McAngus Todd and Merker 2004), the lemur *Indri indri* can reach 110 dB at 0.5 m (sensu Zanoli et al. 2020), titi monkeys at 105 dB (*Plecturocebus discolor*, van Kuijk et al. 2023), and white-handed gibbons sing at 107 dB SPL at 2.7 m (*Hylobates lar*, Terleph et al. 2016). As a reference, white-handed gibbons soft calls, the *hoos,* are emitted at around 25 dB (sensu Clarke et al. 2015), and the softest sound level of the human voice in subjects with a normal, healthy voice is 40–60 dB A at 5 cm (Šrámková et al. 2015). In birds, the yellow-breasted boubou (*Laniarius atroflavus*) sings duet songs that reach 90–103 dB SPL at 1 m (Wheeldon et al. 2021), while banded wren around 90 dB at 1 m (*Thryophilus pleurostictus*, sensu de Kort et al. 2009). Plain-tailed wrens (*Pheugopedius euophrys*) sing duets with higher amplitude than solo songs (Coleman et al. 2021). The authors suggested that this variation in amplitude might reflect the changes in function between the two vocal displays, as duets seem to be used for mate guarding and territorial defense, differently from solos (Coleman et al. 2021).

Duetting behavior has long attracted substantial interest from researchers interested in animal vocal communication because of its level of complexity. In fact, in duets, notes are not given following a random pattern, but animals fine-tune the timing of their emissions so that calls can be alternated (e.g., eastern whipbird, *Psophodes olivaceus;* Watson 1969) or overlapped (e.g., indris, *I. indri*; De Gregorio et al. 2022b). Interactions between emitters within a communicative process are probably among the most fascinating behaviors in the animal world. The fascination that interactions between emitters arouse in a human observer is because turn-taking and overlapping are crucial elements of human communication (Heldner and Edlund 2010; Levinson 2016). Two interlocutors, engaged in a private or public discussion, may interact in conversations where they exchange large amounts of information. The alternation between one speaker and their overlap during vocal exchanges often shows differences concerning several factors, such as cultural context, established social norms, conversational style, and emotional state (Stivers et al. 2009a, b).

Although turn-taking abilities are considered by many to be one of the capabilities peculiar to the human species (Gamba et al. 2016), numerous studies have shown that non-human species may engage in collective displays in which they emit calls taking turns (Demartsev et al. 2018; Adret 2022; De Gregorio et al. 2022b). Extraordinary examples of these collective displays are duets and choruses we observe in a limited number of species across various orders (Dahlin and Benedict 2014). For instance, it is the case of duetting birds engaging in displays in which they take turns precisely or utterly synchronously (Logue 2006; Mann et al. 2006), but it happens also in crickets (*Leptophyes punctatissima*, Zimmermann et al. 1989), bats (*Diaemus youngi*, Carter et al. 2008), meerkats (*Leptophyes punctatissima,* Demartsev et al. 2018), cetaceans (*Physeter microcephalus*, Schulz et al. 2008), and primates (*Indri indri*, De Gregorio et al. 2019).

Duet songs represent a peculiar case of duet because of their complex structure. The vocal interactions between two emitters are considered duet songs when the emissions take the form of songs, complex vocalizations composed of a series of notes of different types uttered following a hierarchical structure (De Gregorio et al. 2022a). ​​When thinking of animals overlapping their calls and taking turns, a well-known case is singing primates (De Gregorio et al. 2021a, 2023; Raimondi et al. 2023). Singing primates are a small circle of species, sometimes phylogenetically close, sometimes distant, that emit long sequences of notes, often modulated in frequency and structured in phrases (Haimoff 1983; Geissmann 2002; De Gregorio et al. 2022a).

### Why duet?

There is little consensus regarding the evolution and function of duets in primates and other duetting taxa, and most of the suggested hypotheses come from avian studies. Tobias et al. (2016) reported that around 1,800 bird species engage in duet behavior, typically associated with year-round territoriality and long-term individual bonding. Numerous studies and reviews, including those by Hall (2004, 2009) and Wickler (1980), have explored duetting in songbirds. Additionally, studies on various bird families and techniques for studying duet functions have been conducted (Douglas and Mennill 2010). Among the suggested hypotheses for the function of avian duets are the joint defense of a shared resource, pair-bond maintenance, mate guarding (Stokes and Williams 1968), and paternity guarding (Sonnenschein and Reyer 1983).

These hypotheses suggest varying expectations regarding cooperation and conflict among pairs of individuals (Hall 2004). If duets primarily collectively protect a shared resource or prevent a partner from being replaced by intruders of the opposite sex, it implies mutual interest among pair members, indicating a cooperative function. In contrast, the idea that duets are crucial for preventing an individual from being replaced within a partnership suggests self-centered motivations, potentially giving rise to sexual conflicts (Parker 1979; Seddon et al. 2002; Hall 2004). In most avian species, duets were concluded to have multiple functions (Dahlin and Benedict 2014).

The research focused on primate duets suggested that duetting behavior serves many different purposes, from strengthening pair bonds to advertising the presence of the pair to neighbors, to actively defending their territory and/or resources, or as a component of mate guarding (Robinson 1979; Rasoloharijaona et al. 2006; Caselli et al. 2015; Dolotovskaya and Heymann 2022). However, it is unclear if duets have multiple functions depending on the context of emission (as suggested for birds, Dahlin and Benedict 2014) or, rather, the species' ecology and social structure and organization.

### Duetting in primates and their social organization

Of the 522 primate species currently recognized (IUCN 2023), a wide variety uses long-distance calls and loud calls (Wich and Nunn 2002; Delgado 2006), while duetting behavior seems to be restricted to a limited number of species, comprising at least 70 species (Tilson and Tenaza 1976; Kappeler 1997; Méndez-Cárdenas and Zimmermann 2009; De Gregorio et al. 2022a). These primates emit joint vocal displays where two individuals coordinate their calls with a degree of temporal precision (de Reus et al. 2021). However, this number is likely an underestimation as not every member of the same genus showing a similar etho-ecology has been investigated in this sense. For example, the species *Phaner furcifer* was split into four species (Groves 2001), but most of the observations and descriptions on duetting behavior were conducted earlier (e.g., Charles-Dominique and Petter 1980; Kappeler 1997).

Historically, duetting primates have been considered pair living, a type of social organization in which one adult male and one adult female live together and coordinate their activities (Kappeler and van Schaik 2002). This idea comes primarily from the works by Haimoff (1986) and Geissmann and Orgeldinger (2000), who were among the first to suggest a link between duet songs and pair bonds in primates. Nevertheless, recent investigations concluded that many duetting singing primates show a flexible social organization comprising pair living and group living, while others even have group-living as their primary social system. Therefore, the presence of a pair-living social organization is not the rule for duetting species (De Gregorio et al. 2022a). This aspect is exciting in light of the findings by Kappeler and Pozzi (2019), namely that the pair-living social organization is ancestral to the group-living one and represents an evolutionary step between a solitary lifestyle and complex, group-living systems.

If duets evolved in pair-living species first, it is plausible that the functions they had in the first place were adapted to the new social organization, as strictly pair-living species (that are often monogamous) might be more interested in defending the territory/resources from intruders. In contrast, more socially flexible and promiscuous species might need to defend their mate against the risk of extra-pair copulations and/or group takeovers by outsiders.

To understand if the proposed link between social organization (considered as the size and composition of a social unit; Kappeler 2019) and duet function holds, we reviewed the available literature on duetting primate species, searching for information on their alleged function and social organization. Note that pair living is often used as a synonym of monogamous, especially in less recent work, while these two traits represent different components of a social system (Kappeler 2019; Fernandez-Duque et al. 2020). Therefore, we considered the social organization level (who lives with whom) rather than the mating system (who mates with whom). Based on the studies' conclusions on the topic, we considered a species as characterized by one or more social organizations.

We found that various functions were linked to duetting behavior, but these functions were often reported as broad concepts and rarely outlined providing a clear definition. For this reason, we grouped functions in two: functions related to mate defense and mate guarding, and functions related to the territory, such as territorial advertisement and territory/resource defense. Moreover, we divided the results of our research into *quantitative evidence* and *descriptive information* (Table SM1). Controlled experiments such as playbacks offer more robust evidence for a specific duetting function than conclusions drawn by observations in the context of duet emissions. Playback experiments can be used to assess, for example, if duets have a mate defense function: if this is the case, animals should show a stronger response to playback of solos than duets, and, in particular, each sex should respond more aggressively to the simulated presence of animals of the same sex. Moreover, animals should perform duets throughout their territory and not preferentially near boundaries. Conversely, if duets have a territorial function, they should occur near territorial boundaries, and males and females would respond aggressively to playbacks. We should also expect that playbacks of duets elicit a stronger response than playbacks of solos. Nevertheless, given that empirical evidence on duetting function is scarce, we also included qualitative inferences in our overview but in a different paragraph.

## Functions of duets: quantitative evidence

### Pair-living primates

Studies on diurnal and duetting pair-living primates as titi monkeys did not support mate guarding as a function of duetting behavior, but found that pairs used coordinated vocal emissions as joint resource defense (*Plecturocebus cupreus*, Dolotovskaya and Heymann 2022), access to food sources regulation and joint territorial defense (*Callicebus nigrifrons*, Caselli et al. 2014, 2015) and, overall, intergroup communication. In particular, playback experiments conducted by Caselli et al. (2015) showed that black-fronted titi monkeys do not show sex-specific responses to playbacks of solos, but males always respond before females regardless of the stimulus type. Playback studies on *Plecturocebus ornatus* demonstrated how calling behavior and response to neighbors increase the chance of group encounters in the early hours of the day. In turn, these encounters and their resulting spacing pattern define and reinforce the location of boundaries (Robinson 1979) on the one hand; on the other hand, they allow for maintaining resource availability and exclusive access to a mate (Robinson 1981). A role in maintaining intergroup separation has also been found for *Cheracebus lucifer* duetting behavior, elicited by both solo male and duet playbacks (Kinzey and Robinson 1983). A recent study using playback experiments in captive settings verified that female titi monkeys attend to and respond to social signals territorially (*P. cupreus*, Lau et al. 2023). Titi monkeys vocalized less and were more oriented toward the speaker when the playback recording was broadcast than the control recordings, independent of social status. Nonetheless, regardless of the stimulus, both the locomotory and vocal response were influenced by the pairing status: pre-pairing females titi were found to spend more time in locomoting than post-pairing. Likewise, pre-pairing females emitted more trills, while post-pairing ones emitted more long calls. Although not through direct testing, the study provided evidence of territorial behavior in *P. cupreus,* presumably involved in territorial occupancy claims and boundary reinforcement (Lau et al. 2023).

Among the Indridae, the indris (*Indri indri*) are the only species with duetting behavior. They are diurnal pair-living lemurs (Torti et al. 2017; Bonadonna et al. 2019) that emit duet and choruses that serve a territorial function and regulate intergroup spacing (Bonadonna et al. 2020; Spezie et al. 2023), but other functions have not explicitly been tested yet, albeit playback experiments assessed the presence of a dear enemy effect (Spezie et al. 2023).

The agile gibbon (*Hylobates agilis*) is a pair-living lesser ape, based on the work by Mitani (1987) that described the species as monogamous, living in groups composed of the adult pair and their offspring (although genetic analyses were not carried out). Extra-pair copulation has never been reported, but researchers observed extra-pair duetting, which could suggest the possibility of extra-pair copulation (Koda et al. 2012). In this species, duetting behavior was linked to territorial displays and mate defense, as playback of male and female solos elicited vocal and behavioral responses from same-sex individuals (Mitani 1987). Mitani (1985) also conducted playback experiments on the southern gray gibbon (*Hylobates muelleri*) and proposed that duets play a role in intergroup spacing and territory defense.

### Primates with a flexible social organization: pair living and group living

The genus *Tarsius* shows considerable variation in social organization (De Gregorio et al. 2022a). In particular, Gursky's spectral tarsier (*T. spectrumgurskyae*) shows pair bonds and polygyny (Gursky 1995; Gursky-Doyen 2010). In this species, playback experiments linked duetting behavior to male vocal mate guarding (Nietsch 2003): female tarsiers might not repel other females showing interest in their mate calling (Nietsch 2003). It has also been suggested that Gursky's spectral tarsiers defend their territories to defend their pair-mate, not the access to resources (Gursky 2003).

The western black crested gibbon (*Nomascus concolor*) is a highly territorial small ape living in social groups that have both monogamous or polygynous (bi-female) mating systems (Fan et al. 2006). This species emits stereotyped male solo songs or duets between the two sexes. Playback experiments showed a *nasty neighbor* effect in this species, and resident groups had more aggressive responses to simulated individual intruders than pairs (Niu et al. 2023), suggesting that duets may serve a mate-defense function. Previous work on the species showed that neighbor males could be competitors for paternity (Huang et al. 2013; Hu et al. 2018)*.* Moreover, Fan et al. (2007) work does not support the intergroup spacing hypothesis for *N. concolor*, in line with what was proposed by Mitani (1985), namely that morning duets may serve to advertise the presence of an active singer.

White-handed gibbons (*Hylobates lar*) are diurnal duetting primates with a flexible social system showing monogamy, polyandry, and polygyny (Brockelman et al. 1998; Reichard et al. 2012). Playback studies in this species indicated a general territorial response and mate defense, where females reacted strongly to females' solo but not males' solos and pair duets, while males reacted to males' solos and duets but not female solos (Raemaekers and Raemaekers 1985).

Siamangs (*Symphalangus syndactylus*) are Hylobatids showing both monogamy and polyandry (Lappan 2008), and early experiments of forced partner exchange in captivity suggested that duets have a pair-bonding function (Geissmann 1999). However, the author pointed out that it was very likely that other functions were also present, although not tested. More recently, Geissmann et al. (2020) analyzed indicators of pair-bond strength in siamangs and concluded that pair bonds in this species might have a mate-defense function. This evidence is in line with the fact that a disruption of the singing pattern in a siamang population caused the arrival of different male outsiders to contend the mating position (Morino 2021).

## Descriptive information

### Pair-living primates

Work on nocturnal duetting lemurs suggested that duets might have the function of coordinating pair activities for intergroup spacing to signal the joint ownership of a territory, as in the case of the pair-living Milne Edwards' sportive lemur (*Lepilemur edwardsi,* Méndez-Cárdenas and Zimmermann 2009). This idea is in line with the work by Rasoloharijaona and colleagues (2006), who suggested that duetting behavior in this species regulates pair cohesiveness and signals territorial ownership.

​​The lariang tarsier (*Tarsius lariang*) is the only tarsier species for which accurate analysis was conducted to investigate the mating system, resulting in a monogamous one (Driller et al. 2009). In this species, the only indication of the presumed function of duets comes from the observations of Merker and Groves (2006), who defined the loud call of the species as a "territorial duet song." A territorial advertisement role of duetting behavior was also suggested for Dian's tarsier (*Tarsius dentatus*, previously named *Tarsius dianae*; Merker 2006), a nocturnal primate endemic to central Sulawesi that was described as pair living (Merker et al. 2004; Tremble 1993).

For titi monkeys, Moynihan proposed one of the first reports of using songs in *territorial defense* in *Plecturocebus moloch* (1966). In this very early description, the author reported that 'gobbling phrases' of different individuals tend to be nearly wholly synchronized and overlap more than 'dawn songs', for instance, and may be directly involved in proclaiming territorial ownership and, consequently, territorial defense. A few years later, Kinzey et al. (1977) observed that the so-called *dawn calls* and the *group solidarity calls* led to intragroup cohesion in *Cheracebus lucifer.* Although not directly tested through experimental procedures, loud dawn duets have been hypothesized to represent a less-expensive form of mate, resource, and territory defense in *Plecturocebus toppini* (Wright 2013). Souza Mattos and colleagues recently described territorial duets of Parecis Plateau Titi Monkey (*Plecturocebus parecis*). Titi monkeys often emit these vocalizations around 6 a.m., before leaving the sleeping site and in response to calls from neighboring groups (Souza Mattos et al. 2023). For both *Plecturocebus modestus* and *Plecturocebus olallae*, researchers suggested a function of songs in mediating intergroup interactions. In particular, songs supposedly have a role in territorial demarcation activity and resource defense during food scarcity (Martinez and Wallace 2016). Lastly, two recent studies provided evidence that titi monkeys' vocalizations play a role as an intergroup spacing mechanism that involves regular announcement of occupancy of a territory (*P. discolor*: Van Belle et al. 2021) and intergroup communication in general (*P. cupreus;* Lau et al. 2023).

Western hoolock gibbons (*H. Hoolock*) are pair-living small apes (Islam and Feeroz 1992; Ahsan 2000), and the description of vocal behavior suggested that duets mediated intergroup competition for resources as a measure of defense (Ahsan 2000).

### Primates with a mixed social organization: pair living and group living

Pale Fork-marked lemurs (*Phaner pallescens*) are nocturnal, pair-living lemurs (Schülke and Kappeler 2003; Schülke 2005), but some social units can be organized in groups, and researchers observed a male sleeping with two different adult females and their infants, along with vocal exchanges (duets) with both females (Charles-Dominique and Petter 1980; Schülke and Kappeler 2003). This suggests these lemurs' social organization might be flexible, and some authors even defined it as a particular case of "pre-gregarious" social organization (Charles-Dominique and Petter 1980), where female residents seem to tolerate female neighbors but not male ones (Schülke and Kappeler 2003). Specific functions of duets have not yet been tested in this species, but observations on these lemurs' behavior suggested that they convey information on the animal identity and position to the pair member and neighboring individuals (Charles-Dominique and Petter 1980). In particular, duet exchange happens in territorial confrontations between neighboring groups in overlapping zones, creating a "concert that lasts 10–20 min" (Charles-Dominique and Petter 1980).

There is virtually no information on Jatna's Tarsier (*Tarsius supriatnai*), but it seems to live in groups that have both a monogamous and a polygamous mating system. Jatna's tarsiers emit a "territorial duet" at dawn before returning to their sleeping sites (Shekelle 2020). Similarly, the Pygmy tarsier (*Tarsius pumilus*) seems to occur in multi-male, multi-female groups or pairs (Grow and Gursky-Doyen 2010; Merker 2016), and they emit ultrasonic duets when leaving and returning to their sleeping sites (Grow et al. 2016).

In the Mentawai leaf monkey (*Presbytis potenziani*) paired males and females take part in a vocal duet directed toward adjacent groups, with a suggested "intergroup spacing function" (Tilson and Tenaza 1976). Moreover, early observation of Mentawai leaf monkeys led Tilson and Tenaza (1976) to suggest that duets serve to maintain a monogamous pair bond because of their vocal resemblance with that of gibbons, titi monkeys, and duetting birds. Nevertheless, if initially this species was considered strictly monogamous (Tilson and Tenaza 1976), more recent studies reported that it could live in stable one-male, one-female; one-male, multi-female; and multi-male, multi-female groups (Sangchantr 2004). This species may represent a case of high flexibility in social organization among non-human primates due to new selective pressures (Sangchantr 2004).

The yellow-cheeked crested gibbon (*Nomascus gabriellae*) presents a mixed social organization comprising pair living and group living (Kenyon et al. 2011; Barca et al. 2016). An investigation of singing probability during different seasons suggested that resource availability affected singing behavior in this species, assuming that song production is costly and used for territorial defense (Rawson et al. 2009). Similarly, a work on Bornean white-bearded gibbons (*Hylobates albibarbis*) used duets as a means to understand the position and size of their territory, based on the idea coming from studies on other gibbon species, that "duet indicates a mated pair engaged in territorial defense" (Cheyne et al. 2008).

For the White-cheeked gibbons (*Nomascus leucogenys*) evidence of the presence of a polygynous mating system in the wild population is more scanty (Harding 2012), as modern studies reported only one male and one female per group (Bach and Rawson 2011). Nevertheless, studies on captive populations found a sex-ratio bias typical of polygynous species (Margulis et al. 2012). Dooley and Judge (2007) observed vocal behavior during a mate change in a pair of captive White-cheeked gibbons and suggested that duetting behavior may only play a minor role in pair-bond maintenance, being more critical to intergroup relations.

### Primates with a group-living social organization

Different species of the genus *Nomascus* are known to show a polygynous mating system, such as the Cao vit gibbon (*Nomascus nasutus*: Fan et al. 2010) and the Hainan gibbon (*Nomascus hainanus*; Zhenhe et al. 1989; Zhou et al. 2008; Guo et al. 2020). In particular, Hainan gibbons have also been observed in multi-male and multi-female groups, although it is unclear whether this social organization is due to the severe fragmentation of their habitat (Li et al. 2022). The use of playback methods for the Hainan gibbon census was based on the idea that groups would respond to advertise the ownership of a territory (Bryant et al. 2016). Similarly, Ma et al. (2020) found that Cao Vit gibbons sing more in core areas or other locations in their territory than in boundary areas, suggesting that duets advertise the occupancy of the territory rather than defend its boundaries.

## The parallelism with duetting birds

Since most duetting species are monogamous (Hall 2004), we have considered the extent of extra-pair paternity (hereafter EEP) in birds as an indicator of the pair's need, whether it is the male or female, to safeguard their bond against potential intruders or betrayals. Duetting birds in tropical and subtropical regions are more likely to have low levels of EPP than non-duetting birds (Douglas et al. 2012). This is supported by the fact that more than 80% of duetting birds live in tropical or subtropical regions (Farabaugh 1982; Langmore 1998; Slater and Mann 2004) and possess most of the traits typically associated with low levels of EPP, such as non-migratory lifestyle, extended breeding seasons, low levels of divorce, high annual adult survival, long-term pair bonds, and enhanced paternal care (Cramer et al. 2011). Still, levels of EPP vary across species, and we will, in this brief paragraph, investigate whether a correlation between EPP (as a proxy for the social system of birds) and the function of duets might exist.

Indeed, it seems that many duetting bird species with low levels of EPP exhibit cooperative and joint defense of resources, while species with higher levels of EPP are more likely to engage in duets to protect the pair bond (mate guarding) or prevent extra-pair copulation (paternity guarding). Research on duet functions across species reveals significant interspecific variation. For example, in red-backed fairy wrens (*Malurus melanocephalus*), Baldassarre et al. (2016) suggested that duets play a role in mate guarding, and this function could be easily connected to the species' high levels of EPP even if the species is socially monogamous and have lifelong pair-bonded individuals. Notably, within the *Malurus* genus, which is generally known for high levels of both extra-pair copulation and paternity, only the species *Malurus coronatus* shows low levels of EPP and exhibits a cooperative territorial defense function for duets: this aligns with the hypothesis that mate-guarding functions are more common in species with higher EPP rates (Kingma et al. 2009). However, there are exceptions, such as two species of *Hypocnemis* (*peruviana* and *cantator*) and *Thryothorus* (*rufalbus* and *ludovicianus*). Despite their low or absent EPP, they employ duets primarily for mate guarding. Duet function varies according to species-specific factors and the widespread nature of duetting in birds across different geographical regions and complexities.

There is remarkable variability from species to species, even in the same genus: *Thryothorus leucotis* shows a very low EPP (3–4%; Gill and Stutchbury 2006) and, in line with what we expected, a mate-guarding function for their duets. Conversely, two other species of the genus show a different pattern: both *Thryothorus rufalbus* and *ludovicianus* have, respectively, low (Douglas et al. 2012) and none (Haggerty et al. 2001) EPP, but they still showed that the primary function of their duets is mate guarding. Another species for which we do not have any information on the EPP is the Bay wrens (*Thryothorus nigricapillus*). This species exhibits diverse duet forms: females typically initiate the duets but rarely sing solo. For males, duets serve as territorial mate guarding, while females help protect the territory from other intruders, revealing potential conflict between mates (Levin 1996a, b). The work of Levin (1996a, b) suggests that female bay wrens use their songs to defend their territory against other females, while males respond to protect their mates. The findings align with observations on three gibbon species: free-living agile gibbons (*Hylobates agilis*; Mitani 1987), Bornean gibbons (*Hylobates muelleri*; Mitani 1984, 1985), and lar gibbons, (*Hylobates lar;* Raemarkers and Raemarkers 1985), where females initiate duets for same-sex defense, supporting the idea that duetting may have evolved due to males and females pursuing distinct strategies within their coordinated song behavior, despite taxonomic differences. While both birds participate in duets, the second bird's response to the duet initiator elicits the duet itself. A solo performance would only be without the second bird joining in with its partner's song. This perspective, as reviewed by Hall (2004), allows for another categorization of the hypotheses regarding the functions of duetting. These hypotheses can be classified based on whom the second bird signals when it harmonizes with its partner's song, the information it conveys through this interaction, and whether its motivations clash with its partner's. According to the theory of Sonnenschein and Reyer in 1983, in species where females take the lead in initiating duets, females use their songs to attract potential mates, while males respond by using their vocalizations to discourage other males, thereby safeguarding their position as potential fathers (e.g., bay wrens, *Thryothorus nigricapillus*). Conversely, in species where males initiate duets (e.g., *Psophodes olivaceus*), females are hypothesized to respond to their partner's song to repel rival males. This response aims to protect females' access to male care, which could be compromised if the male partner seeks a second mate.

Even though duetting birds have been the subject of many studies on their behavior and communication, there is not extensive literature providing for each species information on the EPP and the function of the duetting behavior (Tab. SM2). We found that two species had a high EPP (*Malurus melanocephalus* and *Laniarus atrococcineus*) and performed duets with a mate-guarding function; eight birds species with low EPP did not have a mate-guarding function in their duets (*Malurus coronatus, Strix aluco*, *Campylorhynchus nuchalis, Thryothorus leucotis, Grallina cyanoleuca, Amazona auropalliata, Furnarius rufus, Asio otus*); other four species of duetting birds had a low level of EPP and still a mate-guarding function (*Hypocnemis peruviana* and *cantator*, *Thryothorus rufalbus,* and *ludovicianus*).

## Discussion

We reviewed the available literature regarding the social organization and duet function of 70 primate species, and we found information only on 28 of them (Table SM1). In primates, duetting behavior likely serves a wide range of functions, depending on the species, the context of emission, sex, age, and status of the vocalizing animals (Torti et al. 2013; Zanoli et al. 2023). For example, although duets occur mostly between pair members, juvenile and subadult individuals duet too (Koda et al. 2013; De Gregorio et al. 2021b), and it is plausible that these kinds of vocal interactions might have different functions from the ones involving the reproductive pair, such as mate guarding and pair-bond strengthening.

We chose to focus on two possible functions of primate duets: on one side, the function related to mate guarding and defense, and on the other, the function related to territory advertisement and territory and resource defence. These two aspects are particularly of interest in the light of the findings of Kappeler and Pozzi (2019), namely that a pair-living social organization is ancestral to a group living one, and therefore, duets should have evolved in pair-living species first and, then, were used by group-living ones. Given the lack of consensus regarding the functions of duetting behavior in non-human primates, we wanted to investigate the link between their social organization and the alleged function of duets. In particular, we explored the possibility that strictly pair-living primates might be more interested in defending their territory/resources from intruders, while more promiscuous species might need to defend their mate against extra-pair copulation and/or takeovers.

**Fig. 2:**
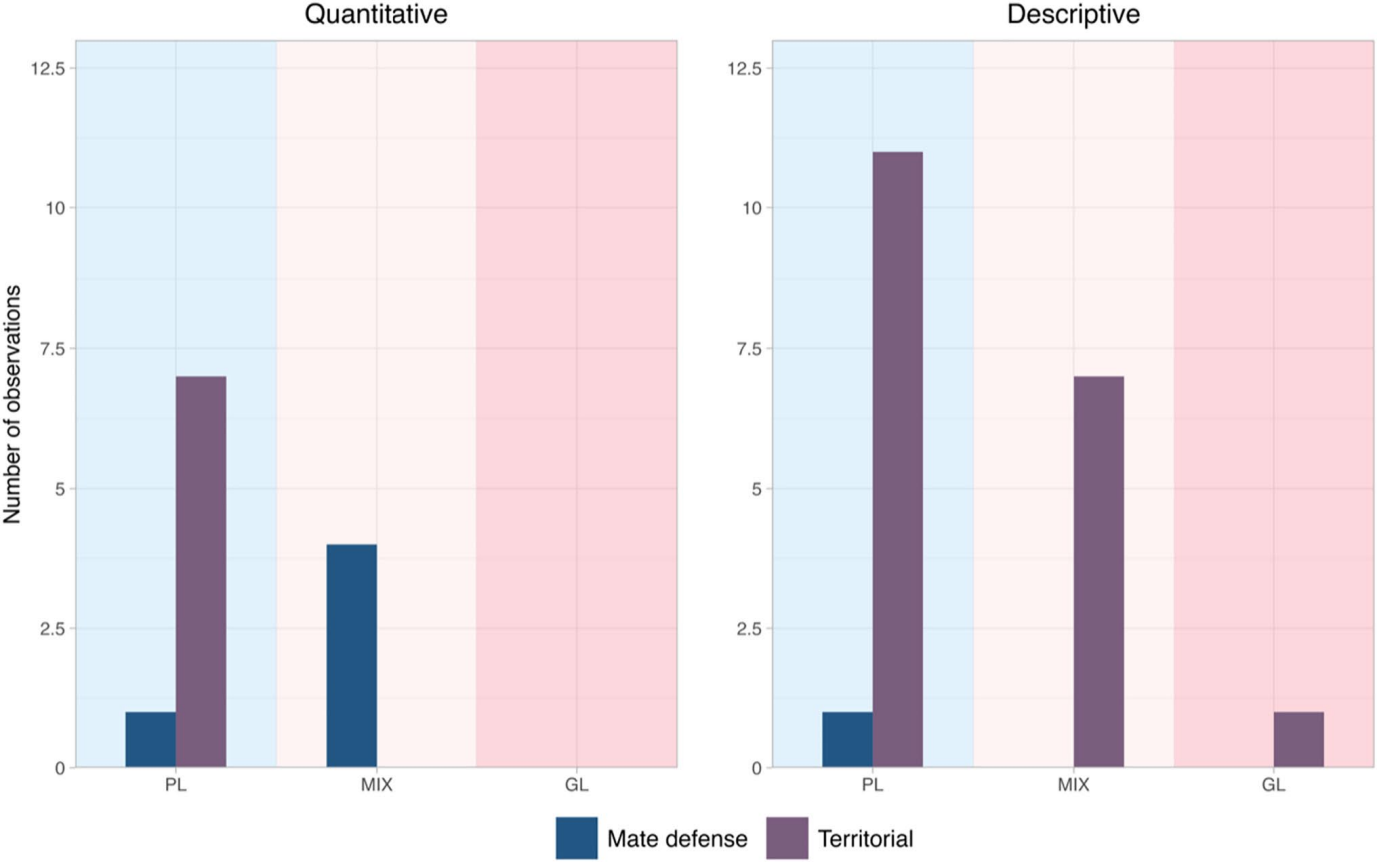
Barplot showing the number of studies (out of 32, Table SM1) that assigned a Mate defense function to primate duets, and the proportion of those that assigned a Territorial function, for each social organization considered (PL: pair living, shaded in light blue; MIX: a species with social units showing both pair living and group living, shaded in light pink; GL: group living, shaded in pink)

The available information was both descriptive and quantitative. In particular, only eleven species of duetting primates have been subjects of studies investigating duetting function with an experimental approach (Table SM1). Most descriptive studies linked duetting behavior to a general territorial function for every social organization considered (pair living, mixed, group living). On the other hand, experimental and quantitative works highlight a critical function of duet in mate defense, especially in primates with a mixed social organization, and more marginally in pair-living ones (Fig. 2), even if our result is based on the only evidence available. Thus, we cannot generalize our conclusions to all duetting primate species. Our work allows a series of considerations.

The first one is that primatologists working on a certain duetting species should not generalize duet functions based on evidence from other species occupying similar ecological niches, as members of the same genus can have different social organizations. For example, gibbons are often presented as having a uniform social organization, but they are more varied at the species and intra-species level regarding group composition and organization. Kappeler and Pozzi (2019), who found that some primate species are obligate pair living while others are facultative pair living, underlined this aspect as many social units contain additional adult members. This is the case for at least 14 species of duetting primates, which we considered to have a mixed social organization between pair living and group living.

The second one is that we can hypothesize that the shift in the social organization from pair living to group living might have led to the emergence of mate defense and mate guarding as an essential function of duetting behavior. This does not mean this function was absent in pair-living species, but it probably played a more marginal role. This idea is also in line with the fact that playback experiments evidence that genetically monogamous species such as indris (Bonadonna et al. 2019) had a dear enemy effect (Spezie et al. 2023), while a more promiscuous species, like the Western crested gibbons, living in both pair and social groups (Fan et al. 2006), had a nasty neighbor effect (Niu et al. 2023). In the Indris, duetting behavior was linked to territorial defense and advertisement, while in Western crested gibbons, it was linked to mate defense. Interestingly, Cowlishaw, in his review of song functions in gibbons (1992), excluded the mate defense hypothesis because he considered gibbons to be monogamous, while nowadays it is well known that monogamy is not the rule in the Hylobatidae family.

Our findings also allow comparison with birds’ duetting behavior. Of the thirty bird species for which we found information on EPP and duetting, we found that most species with low EPP perform duets with a territory and resource defense function, while most species showing high levels of EPP use duetting as a means to guarding their mate (Table SM2). For four species, we found that this was not true, as they show low levels of EPP but their duets still seem to have a mate-guarding function. This might suggest that duet functions are not strictly linked to the social organization, in the case of primates, or the level of EPP, in the case of birds, as we did not find a complete separation in functions depending on the social environment. However, we suggest that social conditions characterized by higher promiscuity are related to the emergence of mate guarding and defense as prominent functions and, in some cases, the only function supported by duetting behavior.

Finally, as Mitani (1985) suggested, functional explanations of duetting behavior should be based on how other animals respond to these vocalizations. Since data on natural interactions between wild resident pairs or groups and solitary individuals are challenging to obtain, controlled experiments such as playbacks are of fundamental importance to imitate such situations, allowing an evaluation of the mate defense and joint territorial/resource defense hypotheses.

We can conclude that, from an empirical perspective, the function(s) of duets remain(s) controversial for three main reasons, however (Hall 2004; Mennill and Vehrencamp 2008). First, conclusions were often drawn from observational studies that lack data on natural interactions between wild resident pairs and solitary individuals. Second, very few studies conducted quantitative analyses to assess the function of these calls. Finally, even fewer studies have tested more than one hypothesis at a time and could, therefore, not offer any comprehensive conclusions. Moreover, for many duetting species, the social organization is still unknown and lacks descriptions of the context in which duets are emitted.

Primates' duetting behavior shows that turn-taking abilities are deeply rooted in human biology and evolution (Levinson 2016), representing the building blocks of human language (Stievers et al. 2009). Our work suggested that, when they evolved first, duets served primarily to regulate access to territory and resources, while they might have assumed a more important role in regulating access to mates with the shift toward a more promiscuous social organization. We encourage future experimental research on this topic, to allow the formulation of empirically testable predictions.

### Supplementary Information

Below is the link to the electronic supplementary material.

Supplementary file1 (DOCX 68 KB)

Supplementary file2 (DOCX 39 KB)

## Data Availability

The information used to create the figures are the tables that are already in the supplement materials.
