# Supplementary material for: Who you live with and what you duet for: a review of the function of primate duets in relation to their social organization
Source: J Comp Physiol A Neuroethol Sens Neural Behav Physiol. 2024 Jan 29;210(2):281–94. doi: 10.1007/s00359-023-01689-9 (PMC10995044; doi:10.1007/s00359-023-01689-9)
Supplement: Supplementary file 1 — Supplementary file1 (DOCX 68 KB) [file 359_2023_1689_MOESM1_ESM.docx]

| **Genus** | **Species** | **Social organization** | **Function (s)** | **PB** | **Descriptive** | **Ref** |
| --- | --- | --- | --- | --- | --- | --- |
| *Indri* | *indri* | SM^[1, 2]^ | Mediate intergroup spatial dynamic |  | **✓** | a |
|  |  |  | Dear enemy effect | **✓** |  | b |
| *Lepilemur* | *edwardsi* | PL ^[3, 4]^ | Joint ownership of a territory |  | **✓** | c, d |
| *Phaner* | *pallescens* | PL, GL ^[5, 6]^ | Position to the pair member and to neighbors |  | **✓** | e |
|  | *dentatus* | PL^[7, 8]^ | Territorial advertisement,  limit hybrid zone | - | **✓** | f, g |
|  | *fuscus* | - | - | - | - | - |
|  | *lariang* | MO^[9]^ | Territorial advertisement,  limit hybrid zone | - | **✓** | g, h |
|  | *niemitzi* | - | - | - | - | - |
|  | *pelengensis* | - | - | - | - | - |
|  | *pumilus* | MM, MF, PL^[10, 11]^ | Leaving and returning to shared sleeping sites and while traveling | - | **✓** | i |
| *Tarsius* | *sangirensis* | PG, MO^[12]^ | - | - | - | - |
|  | *spectrumgurskyae* | PL, PG^[13, 14, 15]^ | Territorial advertisement,  mate attraction/guarding | **✓** | - | j |
|  |  |  | Territorial advertisement | - | **✓** | k |
|  | *supriatnai* | MO, PG^[16]^ | Territorial duet | - | **✓** | l |
|  | *tarsier* | - | - | - | - | - |
|  | *tumpara* | - | - | - | - | - |
|  | *wallacei* | PL, MF^[17]^ | - | - | - | - |
| *Callicebus* | *barbarabrownae* | SM^[18]^ | - | - | - | - |
|  | *coimbrai* | SM^[18]^ | - | - | - | - |
|  | *melanochir* | SM^[18]^ | - | - | - | - |
|  | *nigrifrons* | SM^[18, 19]^ | Access to food sources regulation | **✓** | - | m |
|  |  |  | Joint territorial defense | **✓** | - | n |
|  | *personatus* | SM^[18]^ | - | - | - | - |
| *Cheracebus* | *lucifer* | SM^[18]^ | Inter-group spacing | **✓** | - | o |
|  |  |  | Intra-group cohesion | **-** | **✓** | p |
|  | *lugens* | SM^[18]^ | - | - | - | - |
|  | *medemi* | SM^[18]^ | - | - | - | - |
|  | *purinus* | SM^[18]^ | - | - | - | - |
|  | *regulus* | SM^[18]^ | - | - | - | - |
|  | *torquatus* | SM^[18]^ PL^[20]^ | - | - | - | - |
| *Plecturocebus* | *aureipalatii* | - | - | - | - | - |
|  | *baptista* | SM^[18]^ | - | - | - | - |
|  | *bernhardi* | SM^[18]^ | - | - | - | - |
|  | *brunneus* | SM^[18]^ | - | - | - | - |
|  | *caligatus* | SM^[18]^ | - | - | - | - |
|  | *caquetensis* | - | - | - | - | - |
|  | *cinerascens* | SM^[18]^ | - | - | - | - |
|  | *cupreus* | SM^[18]^ PL^[21]^ GM^[21, 22]^ | Joint territorial defense | **✓** | - | q |
|  |  |  | Inter-group communication (adults) | **-** | **✓** | r |
|  | *discolor* | SM^[18]^ | Inter-group spacing | - | **✓** | s |
|  | *donacophilus* | SM PL^[18]^ | - | - | - | - |
|  | *dubius* | SM^[18]^ | - | - | - | - |
|  | *grovesi* | - | - | - | - | - |
|  | *hoffmannsi* | SM^[18]^ | - | - | - | - |
|  | *miltoni* | - | - | - | - | - |
|  | *modestus* | SM^[18]^ PL^[23]^ | Territorial demarcation | - | **✓** | t |
|  |  |  | Resource defense | - | **✓** | t |
|  | *moloch* | SM PL^[18]^ | Territorial defense | - | **✓** | u |
|  | *oenanthe* | SM^[18]^ PL^[24]^ | - | - | - | - |
|  | *olallae* | SM^[18]^ | Territorial demarcation | - | **✓** | t |
|  |  |  | Resource defense | - | **✓** | t |
|  | *ornatus* | SM PL^[18]^ | Resource defense | **✓** | - | v, w |
|  | *pallescens* | SM^[18]^ | - | - | - | - |
|  | *parecis* | PL^[25]^ | Territorial defense | - | **✓** | x |
|  | *stephennashi* | SM^[18]^ | - | - | - | - |
|  | *toppini* | - | Mate defense,  Territorial defense,  Resource defense | - | **✓** | y |
|  | *urubambensis* | - | - | - | - | - |
|  | *vieirai* | - | - | - | - | - |
| *Presbytis* | *potenziani* | GL^[26]^ | Intergroup spacing |  | **✓** | z |
| *Hoolock* | *hoolock* | MO^[27]^ PB ^[27]^ | Territorial defense,  Territorial advertisement,  Pair-bonding | - | **✓** | aa |
|  | *leuconedys* | ^-^ | - | - | **-** | - |
|  | *tianxing* | MO^[28]^ PB ^[28]^ | - | - | **-** | - |
| *Hylobates* | *abbotti* | - | - | - | **-** | - |
|  | *agilis* | MO^[29]^ PB ^[29]^ | Territorial advertisement,  Resource defense |  | **✓** | ab, ac |
|  |  |  | Territorial defense,  Mate defense | **✓** |  | ad |
|  | *albibarbis* | MO^[30]^ PB ^[30]^ PG^[31]^ | Territorial advertisement,  Intra-group communication | - | **✓** | ae |
|  | *funereus* | SM^[32]^ | Advertisement of male quality | - | **✓** | af |
|  | *lar* | SM^[33]^ PB^[33]^ PA^[34]^ | Territorial advertisement (i.e. spacing)  Pair-bonding |  | **✓** | ag |
|  |  |  | Mate defense,  Territorial defense | **✓** |  | ah |
|  | *muelleri* | MO^[35]^ PB^[35]^ | Territorial advertisement,  Monogamy regulation | **✓** |  | ai |
|  | *pileatus* | MO^[33, 36]^ PG^[36]^ | - | - | **-** | - |
| *Nomascus* | *annamensis* | - | - | - | **-** | - |
|  | *concolor* | PG ^[37]^ | Mate defense,  Pair-bonding,  Resource defense  Group cohesion | - | **✓** | aj, ak |
|  |  |  | Territorial defense,  Territorial advertisement  Mate defense  Resource defense | **✓** | **-** | al |
|  | *gabriellae* | MO^[38]^PG^[39]^ | Territorial advertisement,  Territorial defense | - | **✓** | am |
|  | *hainanus* | G^[40]^ PB^[41]^ | Territorial advertisement,  Inter-group communication | **✓** | **✓** | an |
|  | *leucogenys* | PG^[ 42]^ | Inter-group relation | - | **✓** | ao |
|  | *nasutus* | PG^[43]^ | Territorial advertising | - | **✓** | ap |
|  | *siki* | - | - | - | **-** | - |
| *Symphalangus* | *syndactylus* | MO^[44]^PA^[44]^ | Pair-bonding,  Mate defense | - | **✓** | aq, ar, as |

MO: monogamous; PA: polyandrous; PG: polygynous social system

**Social organization references**

[1] Pollock JI (1975) The social behavior and ecology of *Indri indri*. Dissertation, University College London

[2] Bonadonna G, Torti V, Randrianarison RM, Martinet N, Gamba M, Giacoma C (2014) Behavioral correlates of extra-pair copulation in *Indri indri*. Primates 55:119–123. <https://doi.org/10.1007/s10329-013-0376-0>

[3] Müller AE, Thalmann U (2000) Origin and evolution of primate social organisation: a reconstruction. Biol Rev Camb Philos Soc 75(3):405-435. https://doi.org/10.1111/j.1469-185X.2000.tb00050.x

[4] Rasoloharijaona S, Rakotosamimanana B, Zimmermann E (2000) Infanticide by a male Milne-Edwards' sportive lemur (*Lepilemur edwardsi*) in Ampijoroa, NW-Madagascar. Int J Primatol 21:41-45. <https://doi.org/10.1023/A:1005419528718>

[5] Charles-Dominique P, Petter JJ (1980) Ecology and Social Life of *Phaner furcifer*. In: Charles-Dominique P, Cooper HM, Hladik A, Hladik CM, Pages E, Pariente GF, Petter-Rousseaux A, Schilling A. Nocturnal malagasy primates: ecology, physiology, and behavior. Academic Press, New York, pp 75-95. https://doi.org/10.1016/B978-0-12-169350-3.50008-3

[6] Schülke O, Kappeler PM, Zischler H (2004) Small testes size despite high extra-pair paternity in the pair-living nocturnal primate *Phaner furcifer*. Behav Ecol Sociobiol 55:293-301. <https://doi.org/10.1007/s00265-003-0709-x>

[7] Merker S, Yustian I, Mühlenberg, M (2004) Losing ground but still doing well - *Tarsius dianae* in human-altered rainforests of Central Sulawesi, Indonesia. In: Gerold G, Fremerey M, Guhardja E. Land use, nature conservation and the stability of rainforest margins in Southeast Asia. Springer, Berlin, Heidelberg, pp 299-311. <https://doi.org/10.1007/978-3-662-08237-9_17>

[8] Tremble M (1993) Field observations of *Tarsius dianae* at Lore Lindu National Park, Central Sulawesi, Indonesia. Trop Biodiv 1(2):67-76

[9] Driller C, Perwitasari-Farajallah D, Zischler H, Merker, S (2009) The social system of Lariang tarsiers (*Tarsius lariang*) as revealed by genetic analyses. Int J Primatol 30(2):267-281. <https://doi.org/10.1007/s10764-009-9341-6>

[10] Grow N, Gursky-Doyen S (2010) Preliminary data on the behavior, ecology, and morphology of pygmy tarsiers (*Tarsius pumilus*). Int J Primatol 31:1174-1191. <https://doi.org/10.1007/s10764-010-9456-9>

[11] Merker S (2016) *Tarsius pumilus*. In: Rowe N, Myers M. All the world's primates. Pogonias Press, Charlestown, pp165-166

[12] Shekelle M (2020) *Tarsius sangirensis*. The IUCN Red List of Threatened Species 2020: e.T21493A17977351. <https://dx.doi.org/10.2305/IUCN.UK.2020-3.RLTS.T21493A17977351.en>. Accessed 3 October 2023

[13] Gursky S (1995) Group size and composition in the spectral tarsier, *Tarsius spectrum*: implications for social organization. Trop Biodiv 3(1):57-62.

[14] Gursky-Doyen S (2010) Intraspecific variation in the mating system of spectral tarsiers. Int J Primatol 31:1161-1173. <https://doi.org/10.1007/s10764-010-9450-2>

[15] Gursky S (2000) Sociality in the spectral tarsier, *Tarsius spectrum*. Am J Primatol 51(1):89-101. <https://doi.org/10.1002/(sici)1098-2345(200005)51:1%3C89::aid-ajp7%3E3.0.co;2-7>

[16] Shekelle M (2020) *Tarsius supriatnai* (errata version published in 2022). *The IUCN Red List of Threatened Species* 2020: e.T162336881A220971513. <https://dx.doi.org/10.2305/IUCN.UK.2020-3.RLTS.T162336881A220971513.en>. Accessed 3 October 2023

[17] Merker S (2016) *Tarsius wallacei*. In: Rowe N, Myers M. All the world's primates. Pogonias Press, Charlestown, pp 169

[18] Fernandez‐Duque E, Huck M, Van Belle S, Di Fiore, A (2020) The evolution of pair‐living, sexual monogamy, and cooperative infant care: insights from research on wild owl monkeys, titis, sakis, and tamarins. Am J Phys Anthropol 171(S70):118-173. <https://doi.org/10.1002/ajpa.24017>

[19] Caselli CB, Mennill DJ, Bicca‐Marques JC, Setz EZ (2014) Vocal behavior of black‐fronted titi monkeys (*Callicebus nigrifrons*): Acoustic properties and behavioral contexts of loud calls. Am J Primatol 76(8):788-800. <https://doi.org/10.1002/ajp.22270>

[20] Easley SP, Kinzey WG (1986) Territorial shift in the yellow-handed titi monkey (*Callicebus torquatus*). Am J Primatol 11(4):307–318. <https://doi.org/10.1002/ajp.1350110402>

[21] Ragen BJ, Mendoza SP, Mason WA, Bales KL (2012) Differences in titi monkey (*Callicebus cupreus*) social bonds affect arousal, affiliation, and response to reward. Am J Primatol 74(8):758–769. <https://doi.org/10.1002/ajp.22026>

[22] Dolotovskaya S, Roos C, Heymann EW (2020) Genetic monogamy and mate choice in a pair-living primate. Sci Rep. 10:20328. <https://doi.org/10.1038/s41598-020-77132-9>

[23] Martinez J, Wallace RB (2007) Further notes on the distribution of endemic Bolivian titi monkeys, *Callicebus modestus* and *Callicebus olallae*. Neotrop Primates 14(2):47-54. <https://doi.org/10.1896/044.014.0201>

[24] DeLuycker AM (2012) Insect prey foraging strategies in *Callicebus oenanthe* in Northern Peru. Am J Primatol 74(5):450– 461. <https://doi.org/10.1002/ajp.22002>

[25] Souza Mattos F, de Alencar TB, Boyle SA, Fleck G, Koolen Ferreira HH, Pohlit A, Silva-Diogo O, Gusmão AC, Barnett AA (2023) A life in fragments: the ecology, behavior, and conservation of the recently described parecis plateau titi monkey (*Plecturocebus parecis*). Int J Primatol. <https://doi.org/10.1007/s10764-023-00370-x>

[26] Sangchantr S (2004) Social organization and ecology of Mentawai leaf monkeys (*Presbytis potenziani*). Dissertation, Columbia University

[27] Ahsan MF (2001) Socio-ecology of the hoolock gibbon (*Hylobates hoolock*) in two forests of Bangladesh. In: The apes: challenges for the 21st century. Conference Proceedings, May 10–13, 2000, Chicago Zoological Society, Brookfield, Illinois, U.S.A., pp 286–299

[28] Chan BPL, Mak CF, Yang JH, Huang XY (2017) Population, distribution, vocalization and conservation of the Gaoligong hoolock gibbon (*Hoolock tianxing*) in the Tengchong section of the gaoligongshan national nature reserve, China. Primate Conserv 31:107-113

[29] Mitani J (1987) Territoriality and monogamy among agile gibbons (*Hylobates agilis*). Behav Ecol Sociobiol 20(4): 265–269

[30] Wanelik KM, Azis A, Cheyne SM (2013) Note-, phrase- and song-specific acoustic variables contributing to the individuality of male duet song in the Bornean southern gibbon (*Hylobates albibarbis*). Primates. 54:159–170. <https://doi.org/10.1007/s10329-012-0338-y>

[31] Thompson C, Cahyaningrum E, Birot H, Aziz A, Cheyne SM (2022) A case of polygyny in the Bornean white-bearded gibbon (*Hylobates albibarbis*). Folia Primatol 93(1):97-105. <https://doi.org/10.1163/14219980-20200801>

[32] Inoue Y, Sinun W, Okanoya K (2021) Increase in social interactions of wild Northern Gray gibbons (*Hylobates funereus*) during the mast fruiting period in the Danum Valley Conservation Area, Sabah, Malaysia. Acta Ethol 24:153–163. <https://doi.org/10.1007/s10211-021-00370-1>

[33] Guan ZH, Ma CY, Fei HL, Huang B, Ning WH, Ni QY, Jiang XL, Fan P (2018) Ecology and social system of northern gibbons living in cold seasonal forests. Zool Res 39(4):255-265. <https://doi.org/10.24272/j.issn.2095-8137.2018.045>

[34] Reichard UH (1995) Extra-pair copulations in a monogamous gibbon (*Hylobates lar*). Ethology 100(2):99-112. <https://doi.org/10.1111/j.1439-0310.1995.tb00319.x>

[35] Mitani JC (1984) The behavioral regulation of monogamy in gibbons (*Hylobates muelleri*). Behav Ecol Sociobiol 15(3):225–229. <http://www.jstor.org/stable/4599723>

[36] Srikosamatara S, Brockelman WY (1987) Polygyny in a group of Pileated gibbons via a familial route. Int J Primatol. 8:389–393. <https://doi.org/10.1007/BF02737390>

[37] Fan PF, Jiang XL, Liu CM, Luo WS (2006) Polygynous mating system and behavioural reason of black crested gibbon (*Nomascus concolor jingdongensis*) at Dazhaizi, Mt. Wuliang, Yunnan, China. Zool Res. 27(2):216–220

[38] Kenyon M, Roos C, Binh VT, Chivers D (2011) Extrapair paternity in golden-cheeked gibbons (*Nomascus gabriellae*) in the secondary lowland forest of Cat Tien National Park, Vietnam. Folia Primatol 82:154-164. <https://doi.org/10.1159/000333143>

[39] Barca B, Vincent C, Soeung K, Nuttall M, Hobson K (2016) Multi-female group in the southernmost species of *Nomascus*: field observations in eastern Cambodia reveal multiple breeding females in a single group of southern yellow-cheeked crested gibbon *Nomascus gabriellae*. Asian Primates J 6(1):15-19

[40] Zhou J, Wei F, Li M, Zhang J, Wang D, Pan R (2005) Hainan black-crested gibbon is headed for extinction. Int J Primatol 26:453– 465. <https://doi.org/10.1007/s10764-005-2933-x>

[41] Guo Y, Chang J, Han L, Liu T, Li G, Garber PA, Xiao N, Zhou J (2020) The genetic status of the critically endangered Hainan Gibbon (*Nomascus hainanus*): a species moving toward extinction. Front Genet 11:608633. <https://doi.org/10.3389/fgene.2020.608633>

[42] Harding LE (2012) *Nomascus leucogenys* (Primates: Hylobatidae). Mamm Species 44(890):1–15. <https://doi.org/10.1644/890.1>

[43] Fan P, Fei H, Xiang Z, Zhang W, Ma C, Huang T (2010) Social structure and group dynamics of the Cao Vit gibbon (*Nomascus nasutus*) in Bangliang, Jingxi, China. Folia Primatol 81:245-53. <https://doi.org/10.1159/000322351>

[44] Lappan S (2008) Male care of infants in a siamang (*Symphalangus syndactylus*) population including socially monogamous and polyandrous groups. Behav Ecol Sociobiol 62:1307–1317. <https://doi.org/10.1007/s00265-008-0559-7>

**Functions References**

**a** Bonadonna G, Zaccagno M, Torti V, Valente D, De Gregorio C, Randrianarison RM, Tan C, Gamba M, Giacoma C (2020) Intra- and intergroup spatial dynamics of a pair-living singing primate, *Indri indri*: a multiannual study of three indri groups in Maromizaha forest, Madagascar. Int J Primatol 41:224-245. <https://doi.org/10.1007/s10764-019-00127-5>

**b** Spezie G, Torti V, Bonadonna G, De Gregorio C, Valente D, Giacoma C, Gamba M (2023) Evidence for acoustic discrimination in lemurs: A playback study on wild indris *Indri indri*. Cu Zool 69(1):41–49. <https://doi.org/10.1093/cz/zoac009>

**c** Rasoloharijaona S, Randrianambinina B, Braune P, Zimmermann E (2006) Loud calling, spacing, and cohesivenessin a nocturnal primate, the Milne Edwards’ sportive lemur (*Lepilemur edwardsi*). Am J Phys Anthropol 129(4):591-600. <https://doi.org/10.1002/ajpa.20342>

**d** Méndez-Cárdenas MG, Zimmermann E (2009) Duetting—A mechanism to strengthen pair bonds in a dispersed pair-living primate (*Lepilemur edwardsi*)? AJBA 139(4):523-532. <https://doi.org/10.1002/ajpa.21017>

**e** Charles-Dominique P, Petter JJ (1980) Ecology and Social Life of *Phaner furcifer*. In: Charles-Dominique P, Cooper HM, Hladik A, Hladik CM, Pages E, Pariente GF, Petter-Rousseaux A, Schilling A. Nocturnal malagasy primates: ecology, physiology, and behavior. Academic Press, New York, pp 75-95. https://doi.org/10.1016/B978-0-12-169350-3.50008-3

**f** Merker S (2006) Habitat‐specific ranging patterns of Dian's tarsiers (*Tarsius dianae*) as revealed by radiotracking. Am J Primatol 68(2):111-125. <https://doi.org/10.1002/ajp.20210>

**g** Bohr YE-MB, Purbatrapsila A, Perwitasari-Farajallah D, Ganzhorn JU, Merker S (2023) Strange tunes—acoustic variation and character displacement in a tarsier hybrid zone. Int J Primatol 44:58-612. <https://doi.org/10.1007/s10764-023-00351-0>

**h** Merker S, Groves CP (2006) *Tarsius lariang*: A new primate species from western central Sulawesi. Int J Primatol 27:465-485. <https://doi.org/10.1007/s10764-006-9038-z>

**i** Grow N, Bailey K, Gursky S (2016) Ultrasonic vocalizations by montane pygmy tarsiers, *Tarsius pumilus*. American Association of Physical Anthropologists Conference, Atlanta. <http://dx.doi.org/10.13140/RG.2.1.2208.8089>

**j** Nietsch A (2003) Outline of the vocal behavior of *Tarsius spectrum*: Call features, associated behaviors, and biological functions. In: Tarsiers: Past, present, and future. Rutgers University Press, New Brunswick, pp196-220

**k** MacKinnon J, MacKinnon K (1980) The behavior of wild spectral tarsiers. Int J Primatol 1:361-379. <https://doi.org/10.1007/BF02692280>

**l** Shekelle M (2020) *Tarsius supriatnai* (errata version published in 2022). *The IUCN Red List of Threatened Species* 2020: e.T162336881A220971513. <https://dx.doi.org/10.2305/IUCN.UK.2020-3.RLTS.T162336881A220971513.en>. Accessed 3 October 2023

**m** Caselli CB, Mennill DJ, Bicca-Marques JC, Setz EZ (2014) Vocal behavior of black-fronted titi monkeys (*Callicebus nigrifrons*): acoustic properties and behavioral contexts of loud calls. Am J Primatol 76(8):788-800. <https://doi.org/10.1002/ajp.22270>

**n** Caselli CB, Mennill DJ, Gestich CC, Setz EZ, Bicca-Marques JC (2015) Playback responses of socially monogamous black-fronted titi monkeys to simulated solitary and paired intruders. Am J Primatol. 77(11):1135-42. <https://doi.org/10.1002/ajp.22447>

**o** Kinzey WG, Robinson JG (1983) Intergroup loud calls, range size, and spacing in *Callicebus torquatus*. Am J Phys Anthropol 60(4):539–544. <https://doi.org/10.1002/ajpa.1330600416>

**p** Kinzey WG, Rosenberger AL, Heisler PS, Prowse DL, Trilling JS (1977) A preliminary field investigation of the yellow handed titi monkey, *Callicebus torquatus torquatus,* in Northern Peru. Primates 18(1):159–181. <https://doi.org/10.1007/BF02382957>

**q** Dolotovskaya S, Heymann EW (2022) Coordinated singing in coppery titi monkeys (*Plecturocebus cupreus*): resource or mate defense? Front Ecol Evol Sec. Behavioral and Evolutionary Ecology 10:898509. <https://doi.org/10.3389/fevo.2022.898509>

**r** Lau AR, Clink DJ, Bales KL (2020) Individuality in the vocalizations of infant and adult coppery titi monkeys (*Plecturocebus cupreus*). Am J Primatol 82:e23134. <https://doi.org/10.1002/ajp.23134>

**s** Van Belle S, Porter AM, Fernandez-Duque E, Di Fiore A (2021) Ranging behavior and the potential for territoriality in pair-living titi monkeys (*Plecturocebus discolor*). Am J Primatol 83(5):e23225. <https://doi.org/10.1002/ajp.23225>

**t** Martinez J, Wallace RB (2016) Ecological and behavioural factors influencing territorial call rates for the bolivian titi monkeys, *Plecturocebus modestus* and *Plecturocebus olallae*. Folia Primatol 87:279–290. <https://doi.org/10.1159/000448710>

**u** Moynihan M (1966) Communication in the Titi monkey, *Callicebus*. J Zool 150(1):77-127. <https://doi.org/10.1111/j.1469-7998.1966.tb02999.x>

**v** Robinson JG (1979) Vocal regulation of use of space by groups of titi monkeys *Callicebus moloch.* Behav Ecol Sociobiol 5:1–15. 4599213

**w** Robinson JG (1981) Vocal regulation of inter- and intragroup spacing during boundary encounters in the titi monkey, *Callicebus moloch*. Primates 22(2):161–172. <https://doi.org/10.1007/BF02382607>

**x** Souza Mattos F, de Alencar TB, Boyle SA, Fleck G, Koolen Ferreira HH, Pohlit A, Silva-Diogo O, Gusmão AC, Barnett AA (2023) A life in fragments: the ecology, behavior, and conservation of the recently described parecis plateau titi monkey (*Plecturocebus parecis*). Int J Primatol. <https://doi.org/10.1007/s10764-023-00370-x>

**y** Wright PC (2013) *Callicebus* in Manu National Park: Territory, resources, scent marking and vocalizations. In: Barnett AA, Veiga LM, Ferrari SF, Norconk MA. Evolutionary biology and conservation of Titis, Sakis and Uacaris. Cambridge University Press, Cambridge, pp 232–239. <https://doi.org/10.1017/CBO9781139034210.027>

**z** Tilson RL, Tenaza RR (1976) Monogamy and duetting in an Old World monkey. Nature 263:320-321. <https://doi.org/10.1038/263320a0>

**aa** Ahsan MF (2001) Socio-ecology of the hoolock gibbon (*Hylobates hoolock*) in two forests of Bangladesh. In: The Apes: Challenges for the 21st Century. Conference Proceedings, May 10–13, 2000, Chicago Zoological Society, Brookfield, Illinois, U.S.A., pp 286–299

**ab** Haimoff EH (1984). The organization of song in the agile gibbon (Hylobates agilis). Folia Primatol., 42(1), 42-61. https://doi.org/10.1159/000156143

**ac** Terleph TA, Malaivijitnond S, Reichard UH (2018) An analysis of white-handed gibbon male song reveals speech-like phrases. Am J Phys Anthropol 166(3):649–660. <https://doi.org/10.1002/ajpa.23451>

**ad** Mitani JC (1987) Territoriality and monogamy among agile gibbons (*Hylobates agilis*). Behavior Ecol Sociobiol 20(4):265-269. <https://www.jstor.org/stable/4600018>

**ae** Cheyne SM, Thompson CJH, Phillips AC, Hill RMC, Limin SH (2007) Density and population estimate of gibbons (*Hylobates* *albibarbis*) in the Sabangau Catchment, Central Kalimantan, Indonesia. Primates 49(1):50-56. <https://doi.org/10.1007/s10329-007-0063-0>

**af** Inoue Y, Sinun W, Okanoya K (2021) Increase in social interactions of wild Northern Gray gibbons (*Hylobates funereus*) during the mast fruiting period in the Danum Valley Conservation Area, Sabah, Malaysia. Acta Ethol 24:153–163. <https://doi.org/10.1007/s10211-021-00370-1>

**ag** Terleph T, Saralamba C, Reichard UH (2022) Long-Distance Vocal Signaling in White-Handed Gibbons (*Hylobates lar*). Int J Primatol 43:965–986. <https://doi.org/10.1007/s10764-022-00312-z>

**ah** Raemaekers JJ, Raemaekers PM (1985) Field playback of loud calls to gibbons (*Hylobates lar*): territorial, sex-specific and species-specific responses. Anim Behav 33(2):481-493. <https://doi.org/10.1016/S0003-3472(85)80071-3>

**ai** Mitani JC (1985) Responses of gibbons (*Hylobates muelleri*) to self, neighbor, and stranger song duets. Int J Primatol 6(2):193–200. <https://doi.org/10.1007/BF02693653>

**aj** Fan PF, Xiao W, Huo S, Jiang XL (2009) Singing behavior and singing functions of black-crested gibbons (*Nomascus concolor* *jingdongensis*) at Mt. Wuliang, central Yunnan, China. Am J Primatol 71(7):539-547. <https://doi.org/10.1002/ajp.20686>

**ak** Fan PF, Ni QY, Sun GZ, Huang B, Jiang XL (2008) Seasonal variations in the activity budget of *Nomascus concolor jingdongensis*

at Mt. Wuliang, Central Yunnan, China: effects of diet and temperature. Int J Primatol 29:1047-1057. <https://doi.org/10.1007/s10764-008-9256-7>

**al** Niu X, Guan Z, Ning W, Li X, Sun G, Ni Q, Liu G, Jiang X (2023) Experimental evidence for nasty neighbour effect in western black crested gibbons (*Nomascus concolor*). Behav Ecol Sociobiol 77, 33. <https://doi.org/10.1007/s00265-023-03309-7>

**am** Rawson BM, Clements T, Hor NM (2009) Status and conservation of yellow-cheeked crested gibbons (*Nomascus gabriellae*) in the Seima Biodiversity Conservation Area, Mondulkiri Province, Cambodia. In: Whittaker D, Lappan S. The gibbons: developments in primatology: progress and prospects. Springer, New York. <https://doi.org/10.1007/978-0-387-88604-6_18>

**an** Bryant JV, Brulé A, Wong MHG, Hong X, Zhou Z, Han W, Jeffree TE, Turvey ST (2016) Detection of a new Hainan gibbon (*Nomascus hainanus*) group using acoustic call playback. Int J Primatol 37:534–547. <https://doi.org/10.1007/s10764-016-9919-8>

**ao** Dooley H, Judge D (2007) Vocal responses of captive gibbon groups to a mate change in a pair of white-cheeked gibbons (*Nomascus leucogenys*). Folia Primatol 78(4):228–239. <https://doi.org/10.1159/000102318>

**ap** Ma H, Ma C, Fei H, Yang L, Fan P (2020) Cao Vit Gibbons (*Nomascus nasutus*) sing at higher elevation but not in peripheral areas of their home range in a Karst forest. Int J Primatol 41:701–713. <https://doi.org/10.1007/s10764-020-00178-z>

**aq** Geissmann T, Orgeldinger (2000) The relationship between duet songs and pair bonds in siamangs, *Hylobates syndactylus*. Anim Behav 60(6):805-809. <https://doi.org/10.1006/anbe.2000.1540>

**ar** Geissmann T (1986) Mate change enhances duetting activity in the siamang gibbon (*Hylobates syndactulus*). Behaviour, 96(1/2):17–27. <http://www.jstor.org/stable/4534494>

**as** Morino L, Pasquaretta C, Sueur C, MacIntosh AJJ (2021) Communication network reflects social instability in a wild siamang (*Symphalangus syndactylus*) population. Int J Primatol 42:618–639. <https://doi.org/10.1007/s10764-021-00227-1>
