# Supplementary material for: Who you live with and what you duet for: a review of the function of primate duets in relation to their social organization
Source: J Comp Physiol A Neuroethol Sens Neural Behav Physiol. 2024 Jan 29;210(2):281–94. doi: 10.1007/s00359-023-01689-9 (PMC10995044; doi:10.1007/s00359-023-01689-9)
Supplement: Supplementary file 2 — Supplementary file2 (DOCX 39 KB) [file 359_2023_1689_MOESM2_ESM.docx]

**Table SM2.** Overview of duetting species, their social organization, the presence ✓ or absence ✗ of different functions (MG: mate guarding; PBM: pair-bond mantainance; JRD: joint resource defense) and information on the sex initiating duetting behavior.

| **Genus** | **Species** | **Social organization - EPP** | **MG** | **PBM** | **JRD** | **Start** |
| --- | --- | --- | --- | --- | --- | --- |
| ***Agelaius*** | ***assimilis*** | Socially monogamous ^1-A^ | - | ✓ ^1-B^ | - | **♂**♀ ^1-B^ |
|  | ***phoeniceus*** | Socially polygynous ^1-C^ | do not have duetting behavior | | | |
| ***Amazona*** | ***auropalliata*** | low EPP ^2-A^ | - | **-** | ✓ ^2-B^ | ♂♀^2-B^ |
| ***Asio*** | ***otus*** | no EPP ^3-A^ | - | ✓^3-B^ | ✓^3-B^ | - |
| ***Callaeas*** | ***wilsoni*** | Monogamous year round ^4-A^ | - | - | ✓ ^4-B^ | - |
| ***Campylorhynchus*** | ***nuchalis*** | low EPP (10%) ^5-A^ | - | - | ✓ ^5-B^ | - |
|  | ***rufinucha*** | cooperative breeding ^5-C^ | ✗ ^5-D^ | ✓ ^5-D^ | ✓ ^5-D, E^ | - |
| ***Furnarius*** | ***rufus*** | low EPP ^6-A^ | ✗ ^6-A^ | **-** | ✓ ^6-A^ | ♀^6-B^ |
| ***Grallina*** | ***cyanoleuca*** | low EPP (3-6%) ^7-A^ | ✗ ^7-A, C^ | **-** | ✓^7-B, C^ | ♂♀^7-A^ |
| ***Hypocnemis*** | ***cantator*** | low EPP ^8-A^ | ✓ ^8-B^ | - | - | **♂** ^8-B^ |
|  | ***peruviana*** | low EPP ^8-C^ | ✓ ^8-D^ | - | - | - |
| ***Laniarius*** | ***aethiopicus*** | no info | ✓ ^9-A^ | - | ✓ ^9-A^ | - |
|  | ***atrococcineus*** | high EPP ^9-B^ | ✓ ^9-C^ | ✗ ^9-C^ |  | ♂^9-B^ |
|  | ***atroflavus*** | Socially monogamous^9-D^ | - | - | ✓ ^9-E^ | ♂ ^9-F^ |
|  | ***funebris*** | ? | ✓ ^9-F^ |  | ✓ ^9-F^ |  |
| ***Malurus*** | ***cyaneus*** | high EPP (72%) ^10-A^ | ✓ ♀ | ? | | |
|  | ***coronatus*** | low EPP (4%) ^10-B^ | - | ✓^10-C^ | ✓ ^10-D^ | **♂** ^10-D^ |
|  | ***melanocephalus*** | high EPP (50%) ^10-E^ | ✓^10-F^ | - | ✓ ^10-G^ | - |
| ***Monias*** | ***benschi*** | promiscuous ^11-A^ | ✓^11-B^ | **-** | - | ♀^11-C^ |
| ***Myrmeciza*** | ***longipes*** | ‘Year-long pair bonds’ ^12-A^ | ✗ ^12-B^ | - | ✗ ^12-B^ | - |
| ***Pheugopedius*** | ***fasciatoventris*** | Socially monogamous ^13-A, B^ | - | - | ✓ ^13-A, B^ | - |
| ***Pipilo*** | ***crissalis*** | high EPP (42%) ^14-A^ | - | **-** | ✓^14-B^ | - |
| ***Plocepasser*** | ***mahali*** | moderate EPP (12%) ^15-A^ | - | - | ✓^15-B^ | - |
| ***Psophodes*** | ***olivaceus*** | low rates of pair divorce ^16-A^ | ✓ ^16-B, C^ | ✓ ^16-B, C^ | ✗ ^16-B, C^ | **♂** ^16-D^ |
| ***Strix*** | ***aluco*** | very low EPP ^17-A^ | - | - | ✗ ^17-B^ | - |
| ***Thryothorus*** *(now Thryophilus*) | ***leucotis*** | low EPP (3-4%) ^18-A^ | ✗ ^18-B^ |  |  |  |
|  | ***ludovicianus*** | no EPP ^18-C^ | ✓^18-D^ | **-** | - | **-** |
|  | ***modestus zeledoni*** | **?** | **-** | **-** | ✓ ^18-E^ | **-** |
|  | ***nigricapillus*** | not long lasting bonds | ✓^18-F, G^ | - | ✓^18-F, G^ | ♀^18-F, G^ |
|  | ***rufalbus*** | very low EPP (2-6 %) ^18-G^ | ✓ ^18-H^ | - | ✓ ^18-H^ | - |

**References Table SM2.**

***Agelaius assimilis***

^1-A^: Orians GH (1985) Blackbirds of the Americas. University of Washington Press, Seattle.

^1-B^: Whittingham LA, Kirkconnell A, Ratcliffe LM (1997) The context and function of duet and solo songs in the red-shouldered blackbird. Wilson Bull 109(2):279-289.

***Agelaius phoenicus***

^1-C^: Whittingham LA, Kirkconnell A, Ratcliffe LM (1992) Differences in song and sexual dimorphism between Cuban and North American red-winged blackbirds *(Agelaius phoeniceus*). The Auk 109(4):928-933. https://doi.org/10.2307/4088178

***Amazona auropalliata***

^2-A^: Wright TF, Rodriguez AM, Fleischer RC (2005) Vocal dialects, sex‐biased dispersal, and microsatellite population structure in the parrot *Amazona* *auropalliata*. Mol Ecol 14(4):1197-1205. https://doi.org/10.1111/j.1365-294X.2005.02466.x

^2-B^: Dahlin CR, Wright TF (2012) Duet function in the yellow‐naped amazon, Amazona auropalliata: Evidence from playbacks of duets and solos. Ethol 118(1):95-105. https://doi.org/10.1111/j.1439-0310.2011.01988.x

***Asio otus***

^3-A^: Lawless SG, Ritchison G, Klatt PH, Westneat DF (1997) The mating strategies of eastern screech-owls: a genetic analysis. The Condor 99(1):213-217. https://doi.org/10.2307/1370242

^3-B^: Klatt PH, Ritchison G (1993) The duetting behavior of eastern screech-owls. Wilson Bull, 483-489.

***Callaelas wilsoni***

^4-A^: Hay JR (1984) The kokako-perspective and prospect. Forest and bird 15(1):6-11.

^4-B^: Molles LE, Waas JR (2006) Are two heads better than one? Responses of the duetting kokako to one-and two-speaker playback. Anim behav, 72(1):131-138. https://doi.org/10.1016/j.anbehav.2005.10.012

***Campylorhynchus nuchalis***

^5-A^: Rabenold PP, Rabenold KN, Piper WH, Haydock J, Zack SW (1990) Shared paternity revealed by genetic analysis in cooperatively breeding tropical wrens. Nature 348(6301):538-540. https://doi.org/10.1038/348538a0

^5-B^: Wiley RH, Wiley MS (1977) Recognition of neighbors' duets by stripe-backed wrens *Campylorhynchus* *nuchalis*. Behaviour 10-34. https://doi.org/10.1163/156853977X00027

***Campylorhynchus* *rufinucha***

^5-C^: Bradley DW, Mennill DJ (2009) Solos, duets and choruses: vocal behaviour of the rufous-naped wren (*Campylorhynchus* *rufinucha*), a cooperatively breeding neotropical songbird. J Ornithol, 150:743-753. https://doi.org/10.1007/s10336-009-0393-3

^5-D^: Bradley DW, Mennill DJ (2009) Strong ungraded responses to playback of solos, duets and choruses in a cooperatively breeding Neotropical songbird. Anim Behav 77(5):1321-1327. https://doi.org/10.1016/j.anbehav.2009.01.037

^5-E^: Bradley DW (2008) The form and function of coordinated vocal signalling in a cooperatively breeding neotropical songbird, the rufous-naped wren (*Campylorhynchus* *rufinucha*). Dissertation, University of Windsor

***Furnarius rufus***

^6-A^: Diniz P, Macedo RH, Webster MS (2019) Duetting correlates with territory quality and reproductive success in a suboscine bird with low extra-pair paternity. The Auk 136(1):1-13. https://doi.org/10.1093/auk/uky004

^6-B^: Diniz P, da Silva EF, Webster MS, Macedo RH (2018) Duetting behavior in a Neotropical ovenbird: sexual and seasonal variation and adaptive signaling functions. J Avian Biol 49(4):e01637. https://doi.org/10.1111/jav.01637

***Grallina cyanoleuca***

^7-A^: Hall ML, Magrath RD (2000) Duetting and mate-guarding in australian magpie-larks (*Grallina* *cyanoleuca*). Behav Ecol Sociobiol 47:180-187. https://doi.org/10.1007/s002650050009

^7-B^: Rogers A, Ferguson J, Harrington H, Mcdowell S, Miller A, Panagos J (2004) Use of stereo duet playback to investigate traditional duet playback methods and mechanisms of cooperative territorial defence in magpie-larks. Behaviour 141(6):741-753. https://doi.org/10.1163/1568539042245169

^7-C^: Mulder RA, Bishop H, Cooper M, Dennis S, Koetsveld M, Marshall J, Saunders BL, Langmore NE (2003) Alternate functions for duet and solo songs in magpie-larks, *Grallina cyanoleuca*. Aust J Zool 51(1):25-30. https://doi.org/10.1071/ZO02060

***Hypocnemis cantator***

^8-A^: Zimmer KJ, Isler ML (2003) Family Thamnophilidae (typical antbirds). In: del Hoyo E, Elliott A, Christie D (eds) Handbook of birds of the world, Lynx Editions, Barcelona, pp 448–681.

^8-B^: Seddon N, Tobias JA (2006) Duets defend mates in a suboscine passerine, the warbling antbird (*Hypocnemis* *cantator*). Behav Ecol 17(1):73-83. https://doi.org/10.1093/beheco/ari096

***Hypocnemis peruviana***

^8-C^: Tobias JA, Gamarra‐Toledo V, García‐Olaechea D, Pulgarin PC, Seddon N (2011) Year‐round resource defence and the evolution of male and female song in suboscine birds: social armaments are mutual ornaments. J Evol Biol 24(10):2118-2138. https://doi.org/10.1111/j.1420-9101.2011.02345.x

^8-D^: Tobias JA, Seddon N (2009) Signal jamming mediates sexual conflict in a duetting bird. Curr Biol 19(7):577-582. https://doi.org/10.1016/j.cub.2009.02.036

***Laniarius aethiopicus***

^9-A^: Grafe TU, Bitz, JH (2004) Functions of duetting in the tropical boubou, *Laniarius aethiopicus*: territorial defence and mutual mate guarding. Anim Behav 68(1):193-201. https://doi.org/10.1016/j.anbehav.2003.11.003

***Laniarius atrococcineus***

^9-B^: van den Heuvel IM, Cherry MI, Klump GM (2014) Crimson-breasted shrike females with extra pair offspring contributed more to duets. Behav Ecol Sociobiol 68:1245-1252. https://doi.org/10.1007/s00265-014-1735-6

^9-C^: van den Heuvel IM, Cherry MI, Klump GM (2013) Individual identity, song repertoire and duet function in the crimson-breasted shrike (*Laniarius* *atrococcineus*). Bioacoustics 22(1):1-15. <https://doi.org/10.1080/09524622.2012.701041>

***Laniarius atroflavus***

^9-E^: Wheeldon A, Szymański P, Budka M, Osiejuk TS (2020) Structure and functions of Yellow-breasted Boubou (*Laniarius atroflavus*) solos and duets. PeerJ 8:e10214. https://doi.org/10.7717/peerj.10214

^9-F^: Wheeldon A, Szymański P, Surmacki A, Osiejuk TS (2021) Song type and song type matching are important for joint territorial defense in a duetting songbird. Behav Ecol 32(5):883-894. https://doi.org/10.1093/beheco/arab030

^9-D^: Fry H (2020). Yellow-breasted Boubou (Laniarius atroflavus). In: del Hoyo J, Elliot A, Sargatal J, Christie DA, de Juana E (eds) Birds of the World, version 1.0. Cornell Lab of Ornithology, Ithaca, NY, USA. <https://doi.org/10.2173/bow.yebbou1.01>

***Laniarius funebris***

^9-F^: Sonnenschein E, Reyer HU (1983) Mate‐guarding and other functions of antiphonal duets in the slate‐coloured boubou (*Laniarius funebris*). Ethology 63(2‐3):112-140. https://doi.org/10.1111/j.1439-0310.1983.tb00083.x

***Malurus cyaneus***

^10-A^: Double MC, Cockburn A (2003) Subordinate superb fairy-wrens (*Malurus cyaneus*) parasitize the reproductive success of attractive dominant males. Proc Royal Soc B 270(1513):379-384. https://doi.org/10.1098/rspb.2002.2261

***Malurus coronatus***

^10-B^: Kingma SA, Hall ML, Segelbacher G, Peters A (2009) Radical loss of an extreme extra-pair mating system. BMC Ecol 9:15. ttps://doi.org/10.1186/1472-6785-9-15

^10-C^: Hall ML, Peters A (2009) Do male paternity guards ensure female fidelity in a duetting fairy-wren? Behav Ecol, 20(1):222-228. https://doi.org/10.1093/beheco/arn139

^10-D^: Hall ML, Peters A (2008) Coordination between the sexes for territorial defence in a duetting fairy-wren. Anim Behav 76(1):65-73. https://doi.org/10.1016/j.anbehav.2008.01.010

***Malurus melanocephalus***

^10-E^: Webster MS, Varian CW, Karubian J (2008) Plumage color and reproduction in the red-backed fairy-wren: why be a dull breeder? Behavl Ecol 19(3):517-524. https://doi.org/10.1093/beheco/arn015

^10-F^: Baldassarre DT, Greig EI, Webster MS (2016) The couple that sings together stays together: duetting, aggression and extra-pair paternity in a promiscuous bird species. Biol Lett 12(2):20151025. https://doi.org/10.1098/rsbl.2015.1025

^10-G^: Dowling J, Webster MS (2016) An experimental test of duet function in a fairy-wren (*Malurus*) with moderate cuckoldry rates. Behav Ecol 27(1):228-236. https://doi.org/10.1093/beheco/arv144

***Monias benschi***

^11-A^: Seddon N (2001) The ecology, communication and conservation of the subdesert mesite *Monias benschi*. Doctoral dissertation, University of Cambridge

^11-B^: Seddon N, Butchart SH, Odling-Smee L (2002) Duetting in the subdesert mesite *Monias* *benschi*: evidence for acoustic mate defence? Behav Ecol Sociobiol 52:7-16. https://doi.org/10.1007/s00265-002-0488-9

^11-C^: Seddon N (2002) The structure, context and possible functions of solos, duets and choruses in the subdesert mesite (*Monias benschi*). Behaviour 139(5):645-676. https://doi.org/10.1163/15685390260136753

***Myrmeciza longipes***

^12-A^: Fedy BC, Stutchbury BJ (2004) Territory switching and floating in white-bellied antbird (*Myrmeciza* *longipes*), a resident tropical passerine in Panama. The Auk 121(2):486-496. https://doi.org/10.1093/auk/121.2.486

^12-B^: Fedy BC, Stutchbury BJ (2005) Territory defence in tropical birds: are females as aggressive as males? Behav Ecol Sociobiol 58:414-422. https://doi.org/10.1007/s00265-005-0928-4

***Pheugopedius fasciatoventris***

^13-A^: Logue DM, Gammon DE (2004) Duet song and sex roles during territory defence in a tropical bird, the black-bellied wren, *Thryothorus fasciatoventris*. Anim Behav 68(4):721-731. https://doi.org/10.1016/j.anbehav.2003.10.026

^13-B^: Logue DM (2007) How do they duet? Sexually dimorphic behavioural mechanisms structure duet songs in the black-bellied wren. Anim Behav 73(1):105-113. https://doi.org/10.1016/j.anbehav.2006.05.011

***Pipilio crissalis***

^14-A^: Benedict L (2008) Unusually high levels of extrapair paternity in a duetting songbird with long-term pair bonds. Behav Ecol Sociobiol 62:983-988. https://doi.org/10.1007/s00265-007-0524-x

^14-B^: Benedict L (2010) California towhee vocal duets are multi-functional signals for multiple receivers. Behaviour 147(8):953-978. https://doi.org/10.1163/000579510X498633

***Plocepasser mahali***

^15-A^: Harrison XA, York JE, Cram DL, Hares MC, Young AJ (2013) Complete reproductive skew within white-browed sparrow weaver groups despite outbreeding opportunities for subordinates of both sexes. Behav Ecol Sociobiol 67:1915-1929. https://doi.org/10.1007/s00265-013-1599-1

^15-B^: Voigt C, Leitner S, Gahr M (2006) Repertoire and structure of duet and solo songs in cooperatively breeding white-browed sparrow weavers. Behaviour 143(2):159-182. https://doi.org/10.1163/156853906775900739

***Psophodes olivaceous***

^16-A^: Rogers AC, Mulder RA (2004) Breeding ecology and social behaviour of an antiphonal duetter, the eastern whipbird (*Psophodes* *olivaceus*). Aust J Zool 52(4):417-435. https://doi.org/10.1071/ZO04001

^16-B^: Rogers AC, Langmore NE, Mulder RA (2007) Function of pair duets in the eastern whipbird: cooperative defense or sexual conflict? Behav Ecol 18(1):182-188. https://doi.org/10.1093/beheco/arl070

^16-C^: Rogers AC, Mulder RA, Langmore NE (2006) Duet duels: sex differences in song matching in duetting eastern whipbirds. Anim Behav 72(1):53-61. https://doi.org/10.1016/j.anbehav.2005.08.019

^16-D^: Rogers AC (2005) Male and female song structure and singing behaviour in the duetting eastern whipbird, *Psophodes* *olivaceus*. Aust J Zool 53(3):157-166. https://doi.org/10.1071/ZO04083

***Strix aluco***

^17-A^: Saladin V, Ritschard M, Roulin A, Bize P, Richner H (2007) Analysis of genetic parentage in the tawny owl (*Strix* *aluco*) reveals extra-pair paternity is low. J Ornithol 148:113-116. https://doi.org/10.1007/s10336-006-0109-x

^17-B^: Appleby BM, Yamaguchi N, Johnson PJ, Macdonald DW (1999) Sex‐specific territorial responses in tawny owls *Strix* *aluco*. Ibis, 141(1):91-99. https://doi.org/10.1111/j.1474-919X.1999.tb04267.x

***Thryothorus leucotis***

^18-A^: Gill SA, Stutchbury BJ (2006) Long-term mate and territory fidelity in neotropical buff-breasted wrens (*Thryothorus* *leucotis*). Behav Ecol Sociobiol 61:245-253. https://doi.org/10.1007/s00265-006-0255-4

^18-B^: Gill SA, Vonhof MJ, Stutchbury BJ, Morton ES, Quinn JS (2005) No evidence for acoustic mate-guarding in duetting buff-breasted wrens (*Thryothorus* *leucotis*). Behav Ecol Sociobiol 57:557-565. https://doi.org/10.1007/s00265-004-0893-3

***Thryothorus ludovicianus***

^18-C^: Haggerty TM, Morton ES, Fleischer RC (2001) Genetic monogamy in Carolina wrens (*Thryothorus* *ludovicianus*). The Auk 118(1):215-219. https://doi.org/10.1093/auk/118.1.215

^18-D^: Zapata D (2022) Intrasexual territoriality, degree of aggression, and duet function in the Carolina Wren (*Thryothorus ludovicianus*). Dissertation, Missouri State University

***Thryothorus modestus zeledoni***

^18-E^: Marshall–Ball L, Slater PJ (2004) Duet singing and repertoire use in threat signalling of individuals and pairs. Proc Royal Soc B 271(Suppl 6):S440-S443. https://doi.org/10.1098/rsbl.2004.0186

***Thryothorus nigricapillus***

^18-F^: Levin RN (1996) Song behaviour and reproductive strategies in a duetting wren, *Thryothorus* *nigricapillus*: II. Playback experiments. Anim Behav 52(6):1107-1117. https://doi.org/10.1006/anbe.1996.0258

^18-G:^ Levin RN (1996) Song behaviour and reproductive strategies in a duetting wren, *Thryothorus nigricapillus*: I. Removal experiments. Anim Behav 52(6):1093-1106. https://doi.org/10.1006/anbe.1996.0257

***Thryothorus rufalbus***

^18-G^: Douglas SB, Heath DD, Mennill DJ (2012) Low levels of extra-pair paternity in a neotropical duetting songbird, the rufous-and-white wren (*Thryothorus* *rufalbus*). The Condor 114(2):393-400. https://doi.org/10.1525/cond.2012.110028

^18-H^: Topp SM, Mennill DJ (2008) Seasonal variation in the duetting behaviour of rufous-and-white wrens (*Thryothorus* *rufalbus*). Behav Ecol Sociobiol 62:1107-1117. <https://doi.org/10.1007/s00265-007-0538->4
